# Treatment of congenital auricular fistula with local skin flaps: Two case reports

**DOI:** 10.1016/j.bjorl.2024.101454

**Published:** 2024-06-06

**Authors:** Makoto Akutsu, Yuni Masuyama, Yuumi Nagashima, Satoru Fukami, Yasuhiro Tanaka, Hideki Hirabayashi, Sin-ichi Haruna

**Affiliations:** a Dokkyo Medical University, Department of Otorhinolaryngology, Head and Neck Surgery, Tochigi, Japan; b Dokkyo Medical University Saitama Medical Center, Department of Otorhinolaryngology, Head and Neck Surgery, Saitama, Japan

## Introduction

Congenital auricular fistula, a congenital anomaly around the auricle, develops owing to the incomplete fusion of hillocks arising from the first and second branchial arches during the embryonic stage. The prevalence of this common congenital auricular anomaly is 1%–2% worldwide, which is lower (0.6%–0.7%) in Japan.^1^

Without a history of auricular fistula infection or swelling in the surroundings, no treatment is required. Antibiotic treatment is administered in cases of confirmed infection. However, infections recur. Thus, surgery (congenital auricular fistula tract removal surgery) is required.^2^ This is a basic technique to identify and remove the auricular fistula from the auricular cartilage. In cases of infection with abscess formation and those of residual skin redness and swelling after antibiotic treatment, removal of infected and contaminated tissue and congenital auricular fistula tract is necessary. However, infections recur even after surgical treatment, making disease control difficult.^3^

We report such severe cases wherein we achieved good progress by extensively excising infected and contaminated tissues and closing wounds using local skin flaps.

## Case report

### Case 1: A 5-year-old girl

#### Progress

Swelling in the right preauricular area was noted in early February 2020. She visited a local otorhinolaryngology clinic the next day, wherein congenital auricular fistula infection was suspected. She was referred to our department on the same day for detailed examination and treatment. The infection improved after treatment with incision, drainage, and oral antibiotics. However, surgical treatment was decided for future infection control; thus, she was admitted in March 2020.

Medical history

Autism.

Allergy

Amoxicillin (skin rashes).

#### Wound findings

**Fig. 1:**
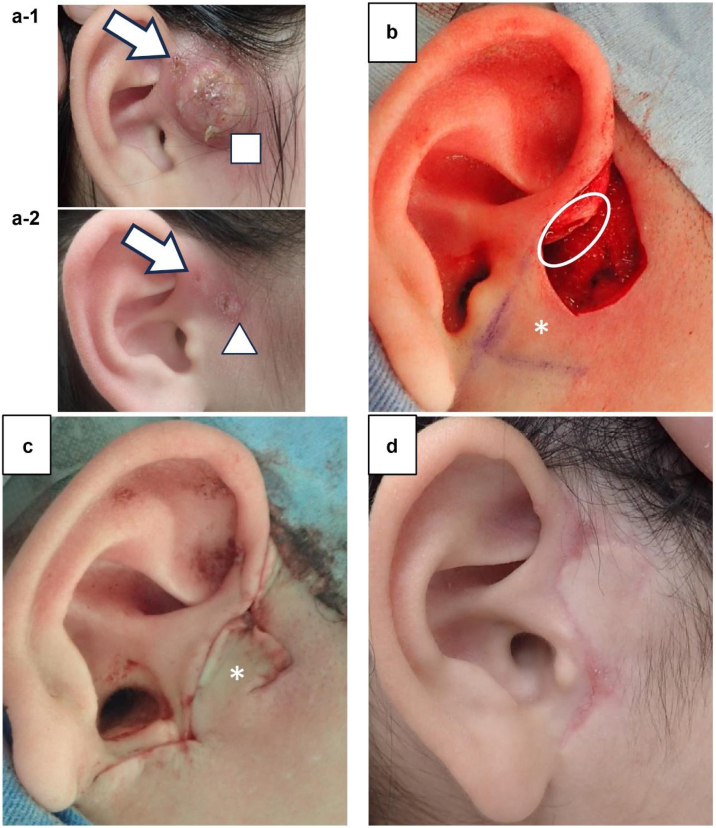
Case 1 (a-1) Findings at the first visit. Abscess formation (□) is observed below the auricular fistula opening (arrow). (a-2) Findings on the day before surgery. The abscess improves with antibiotic treatment, but there is crusting (△) consistent with the area where the abscess is formed. (b) Intraoperative findings. The auricular fistula tract is continuous until the auricular cartilage (○). It is excised with the crusted skin. A skin flap (*) is created below the excision area. (c) Intraoperative findings. The skin flap (*) is rotated, covering the wound. (d) Wound findings 6 months after surgery.

**Fig. 2:**
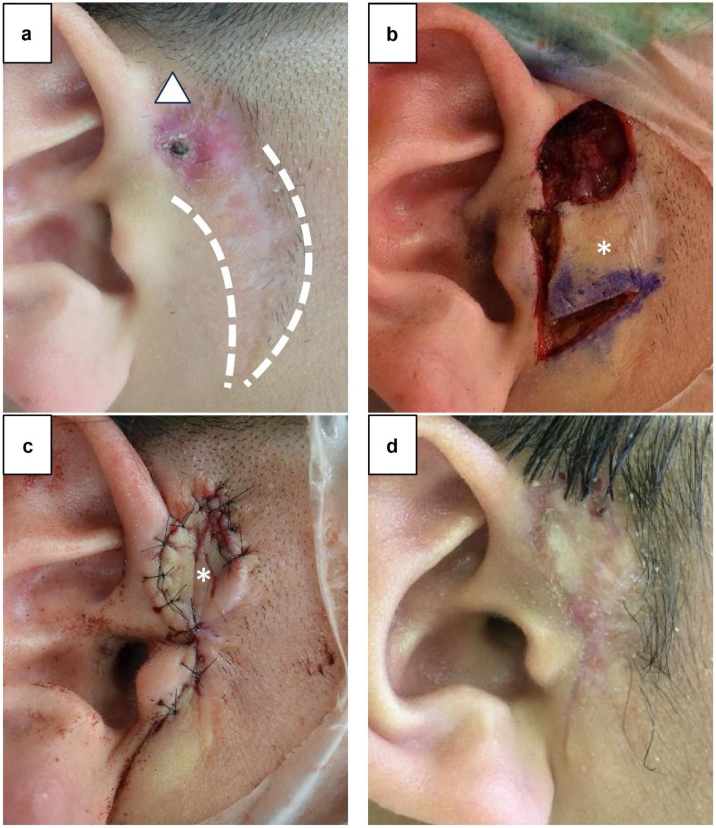
Case 2. (a) Findings on the day before surgery. Skin scarring (dotted line) from the previous surgery is observed, and skin redness with crusting (△) is observed above it. (b) Intraoperative findings. The auricular fistula tract cannot be identified, and reddened skin and subcutaneous contaminated tissues are excised. A skin flap (*) is created below the excision area. (c) Intraoperative findings. The skin flap (*) is rotated, covering the wound. (d) Wound findings 3 months after surgery.

At the first visit, auricular fistula opening was observed at the anterior edge of the helix; skin swelling, and abscess formation were confirmed below it (Fig. 1a-1). Crust adhesion and residual skin redness with skin swelling were observed at admission (Fig. 1a-2).

#### Surgical findings

Surgical treatment was performed under general anesthesia. The auricular fistula tract was identified with a bougie. Despite excising the fistula tract continuously from the auricular fistula, crusting and red areas on the skin were excised. The auricular fistula was based on the cartilage at the anterior edge of the helix. Thus, part of the cartilage in the same area was also excised (Fig. 1b). Since the skin defect area was extensive, the wound could not be closed with simple suturing. Thus, a skin flap was created directly below the excision area. The skin flap was rotated, and the wound was covered and sutured (Fig. 1c).

#### Postoperative progress

The postoperative wound recovered without any problem; no reinfection occurred. The patient was followed up for 6 months after surgery. However, no wound infection was observed (Fig. 1d). There was no wound scarring; good progress was achieved. Thus, the patient was advised to undergo a follow-up at a local clinic.

### Case 2: A 14-year-old boy

#### Progress

In 2015, he underwent congenital auricular fistula tract removal surgery at another hospital. Afterward, as repeated swelling and pain were confirmed around the wound, he visited a local otorhinolaryngology clinic. Residual/infected auricular fistula tract was considered possible. Thus, he was referred to our department in November 2017 for detailed examination and treatment. Surgical treatment was decided upon future infection control, for which he was subsequently admitted in July 2018.

Medical history

Congenital auricular fistula tract removal.

Allergy

None.

Wound findings

Keloid scarring was observed at the wound from the previous surgery, and skin redness with crusting was observed behind it (Fig. 2a).

#### Surgical findings

Surgical treatment was performed under general anesthesia. The opening of the auricular fistula tract was excised during the previous surgery. Additionally, the auricular fistula tract could not be identified on the skin surface. The area with skin redness and contaminated tissues were excised as much as could be visually confirmed (Fig. 2b). Since the skin defect area was extensive, a skin flap was created directly below the tissue excision area. The skin flap was rotated, and the wound was covered and sutured (Fig. 2c).

#### Postoperative progress

The postoperative wound recovered without any complications; no reinfection occurred after surgery. The patient was followed up for 3 months after surgery, wherein the progress was good. Thus, the patient was advised to undergo a follow-up at a local clinic (Fig. 2d).

## Discussion

Providing surgical treatment of congenital auricular fistula, infection control should be emphasized. In particular, regarding simple congenital auricular fistula tract removal surgery, contaminated tissues cannot be excised, and infection foci would remain.

This report showed that cases of extensive contaminated tissues and excision areas could be treated with local skin flaps and that infection control was possible in cases of repeated infections even after auricular fistula tract removal by removing the causative foci of infections. In both cases, we used local skin flaps created close to the excision area and rotated to cover the wound.^4^ In the past, surgery using flap has been reported for congenital auricular fistulas.^5^ Compared with the surgical findings, we suggest that the advantages of management of these two cases are that the skin incision was narrower and less invasive. Moreover, the design of the flap was simpler and more versatile. In addition, the local skin flaps were esthetically advantageous as the flap color tone was similar to that of the excision area.

Skin flaps involve less tension on the wound compared with simple suturing. However, wound dehiscence and keloid scars are concerns as postoperative complications. Therefore, considering skin flap design is necessary. Additionally, surgery time tends to be slightly longer than that for simple removal.

## Conclusion

This surgical procedure for congenital auricular fistula using a local skin flap was effective in cases of abscess formation and repeated infection.

## Conflicts of interest

The authors declare no conflicts of interest.
